# Effects of Restricted Fructose Access on Body Weight and Blood Pressure Circadian Rhythms

**DOI:** 10.1155/2012/459087

**Published:** 2012-03-29

**Authors:** Danielle Senador, Swapnil Shewale, Maria Claudia Irigoyen, Khalid M. Elased, Mariana Morris

**Affiliations:** ^1^Department of Pharmacology & Toxicology, Boonshoft School of Medicine, Wright State University, 3640 Colonel Glenn Highway, Dayton, OH 45435, USA; ^2^Heart Institute, School of Medicine, University of São Paulo, 05403-900 São Paulo, SP, Brazil

## Abstract

High-fructose diet is known to produce cardiovascular and metabolic pathologies. The objective was to determine whether the timing of high fructose (10% liquid solution) intake affect the metabolic and cardiovascular outcomes. Male C57BL mice with radiotelemetric probes were divided into four groups: (1) 24 h water (control); (2) 24 h fructose (F24); (3) 12 h fructose during the light phase (F12L); (4) 12 h fructose during the dark phase (F12D). All fructose groups had higher fluid intake. Body weight was increased in mice on restricted access with no difference in total caloric intake. Fasting glycemia was higher in groups with restricted access. F24 mice showed a fructose-induced blood pressure increase during the dark period. Blood pressure circadian rhythms were absent in F12L mice. Results suggest that the timing of fructose intake is an important variable in the etiology of cardiovascular and metabolic pathologies produced by high fructose consumption.

## 1. Introduction

Given the substantial levels of fructose consumption in our everyday diet, it is important to delineate its consequences [1, 2]. There is compelling evidence that increased fructose intake has metabolic and cardiovascular effects in both human and animals [3–8]. Our group showed that a high-fructose diet in mice produced glucose intolerance and increased blood pressure [4, 6]. Evidence also showed that sympathetic activation occurred rapidly before fructose induction of metabolic dysfunction [9]. A clinical study showed that ingestion of water containing 60 g of fructose acutely elevates blood pressure in healthy human subjects [3].

In addition to the global detrimental effects of high fructose intake, the timing of consumption might also play a causative role in the metabolic and cardiovascular pathologies. There are many examples of work and lifestyle patterns that require alternative intake cycles. This is seen in public service work: police and fire protection, transportation, and utilities, all of which rely on 24 hr around the clock attention. Likewise, shift work as required in health care or manufacturing industries forces people to be active in the normal sleeping phase of the light-dark (L/D) cycle. Obesity and metabolic syndrome are often associated with the nocturnal eating syndrome and shift work, demonstrating the connection between L/D cycle and metabolic pathologies [10–15]. Circadian misalignment has also been shown to produce changes in glucose and insulin levels, as well as increases in blood pressure, in both mice and humans [16–18]. Although the mechanisms underlying these adverse cardiometabolic changes remain unknown, it is possible that disturbances in the sleep/wake and eating schedule, could, in turn, influence appetite, food intake, and energy balance. This could have important implications for the increased obesity, diabetes, and cardiovascular disease in the shift work population [15].

In the context of the worldwide epidemic of obesity and diabetes [19], the increase in sugar consumption in our contemporary western diets [20], and the increase in sedentary lifestyles [21], we conducted studies in mice to evaluate the effects of 24 h access to fructose as well as the influence of the timing of consumption (fructose access provided only in the light or dark period). The parameters of interest focused on cardiovascular and metabolic function: 24 hr L/D rhythm in BP, glucose tolerance, plasma insulin, and the pattern and amount of fructose intake.

## 2. Methods

### 2.1. Animals, Surgery, and General Procedures

 Eight-week-old male C57BL mice (~25 gm, Harlan Inc, Indianapolis, IN) were given water or fructose (10%) for 8 weeks. Groups are (1) 24 h water (Control, *n* = 6); (2) 24 h fructose (F24, *n* = 6); (3) 12 h fructose during the light phase (F12L, *n* = 6); (4) 12 h fructose during the dark phase (F12D, *n* = 6). Animals were housed at 22°C under a 12/12 light/dark cycle with *ad libitum* access to standard pellet chow and water or fructose. Telemetric probes (model TA11PA-C10, Data Sciences International, St. Paul, MN) were inserted into the left common carotid artery at 6 weeks in mice anesthetized with ketamine/xylazine (120 : 20 mg/kg, im). The transmitter body was positioned subcutaneously on the right flank [22, 23]. 24 h cardiovascular recordings, fluid, and food consumption measurements were made at 8 wks. Body weight was measured during the 2nd, 4th, and 8th weeks of the experimental protocol.

### 2.2. Glucose Tolerance Test (GTT)

 GTT was performed at the beginning of the 8th week (last week) of the experimental protocol. Mice were fasted, with animals receiving only water, for 6 h. Blood samples were taken from a tail cut at 0, 15, 30, 60, and 90 min after i.p. glucose load (1.5 g/kg). Blood glucose was determined by Accu-Chek Advantage Blood Glucose Monitor (Roche Diagnostic Corporation, Indianapolis, IN).

### 2.3. Plasma Measurements

 At the end of the 8th week of treatment, mice were sacrificed by decapitation and trunk blood was collected in ice-chilled heparinized tubes. Total plasma cholesterol and triglycerides were determined by colorimetric enzymatic assays (Thermo Fisher Scientific Inc., Waltham, MA). Plasma insulin levels were measured using a multianalytic profiling beads assay (Linco Research Inc., St Charles, MO).

### 2.4. Urinary Corticosterone Measurements

 Spot urine samples (100~100 uL) were collected during the last hour of the light phase. Urinary corticosterone levels were measured by radioimmunoassay (MP Biomedical, Orangeburg, NY, USA). Urinary creatinine was measured by spectrophotometry (Microvue Creatinine Assay Kit, Quidel Corporation, San Diego, USA). The corticosterone : creatinine ratio was calculated for each urine specimen.

### 2.5. Statistics

Values were expressed as mean ± SEM. Data were analyzed using ANOVA two-way or repeated measures when applicable and followed by the Newman-Keuls test. Differences were considered to be statistically significant at *P* < 0.05.

## 3. Results

**Figure 1:**
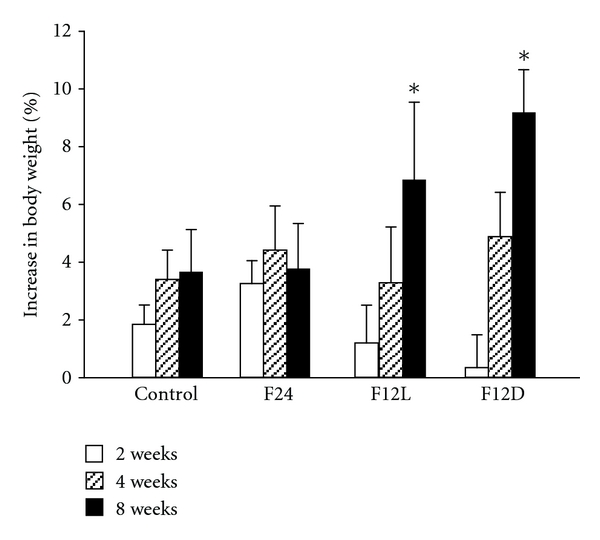
Body weight gain as percentage increase from day 0 in control and fructose-treated groups. ANOVA showed main effect of time (F (2.6156) = 8.97.23, *P* < 0.0012). *P < 0.015 vs. 2 wks.

**Figure 2:**
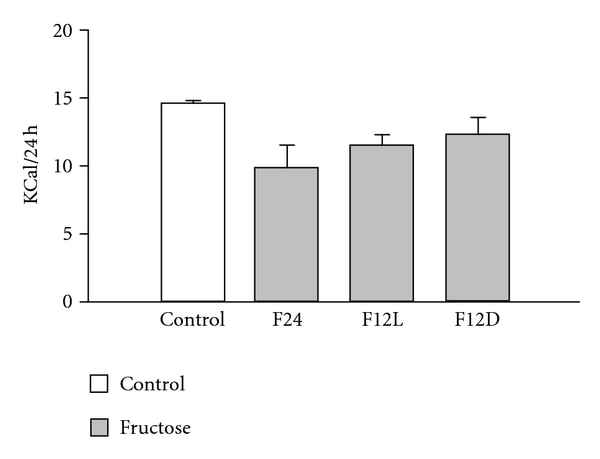
Daily total caloric intake and fructose percentage caloric intake in control and fructose-treated groups.

Baseline body weights (BWs) ranged from 26 to 28 g. The percentage increase in BW over the 8 wk period was significantly higher in the restricted access groups (Figure 1). The F12L and F12D groups showed significant increases in BW gain after 8 wks of treatment as compared to 2 wks (*P* < 0.05). Total caloric intake was not different between groups (Figure 2).

**Figure 3:**
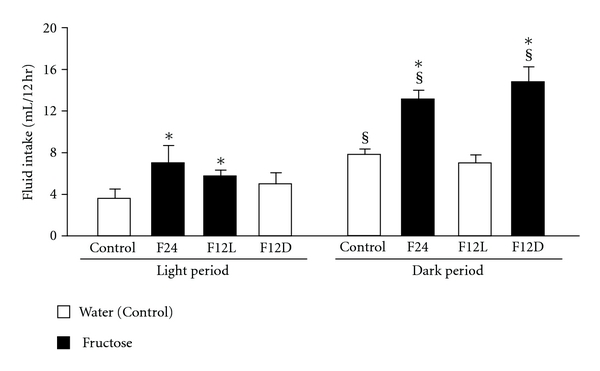
12 h fluid intake in control and fructose groups. ANOVA showed main effect of fructose diet [F (3.20) = 8.4, *P* < 0.001], light/dark (F (1.20) = 50.2, *P* < 0.00001) and interaction between diet and light/dark (F (3.20) = 5.7, *P* < 0.01). ^§^ *P* < 0.01 light vs. dark. *P < 0.01 versus Control.

Fluid intake was measured during the light and dark phases with significant effects of diet, light/dark, and interaction between diet and light/dark (Figure 3). 24h fluid intake was higher in F24 and F12D mice when compared to Control group (*P* < 0.01). There were light/dark differences in intake in Control, F24, and F12D groups. In the F12L group, there was no difference in fluid intake between the light and dark phases. In the restricted access groups, fluid intake was greatest during the fructose consuming period, light for F12L and dark for F12D (*P* < 0.01 vs. Control). With regard to the percentage fluid intake during light and dark phases, mice consuming fructose during the dark period drank a greater percentage of fluid, 67–74%, during this phase (Table 2). Mice given fructose during the light period drank almost equal amounts during the light and dark (44 and 56% resp.).

**Figure 4:**
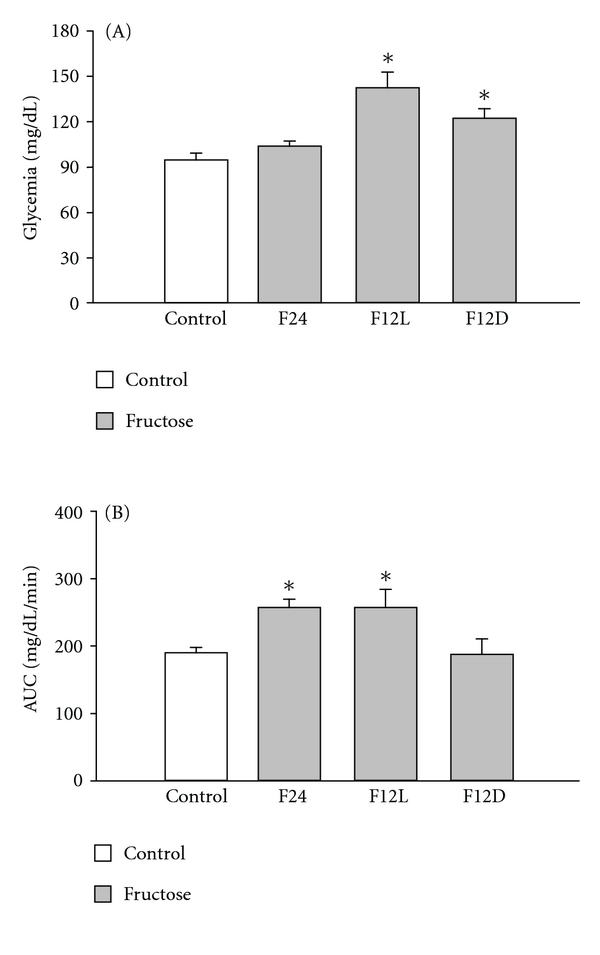
(A) Fasting glycemia in control and fructose groups. F12L and F12D mice showed hyperglicemia ANOVA treatment (F (1.18) = 12.3, *P* < 0.0003). *P < 0.01 versus control. (B) Glucose tolerance test estimated by area under the time curve (AUC) in control and fructose-treated groups. F24 h and F12L mice showed impaired glucose tolerance. ANOVA treatment (F (1.16) = 4.9, *P* < 0.05). *P < 0.01 versus Control.

Fasting glucose was higher in groups under restricted fructose access as compared to Control (Figure 4(A), *P* < 0.01) with higher levels in F12L as compared to F12D (Figure 4(A), *P* < 0.01). GTT showed impaired glucose tolerance in F24 and F12L groups as compared to control (Figure 4(B), *P* < 0.01).

There were no significant changes in insulin levels among groups (Table 1). Triglyceride levels were lower in restricted fructose access groups (Table 1, *P* < 0.01). Both plasma cholesterol and urinary corticosterone levels showed no difference among the groups (Table 1).

**Figure 5:**
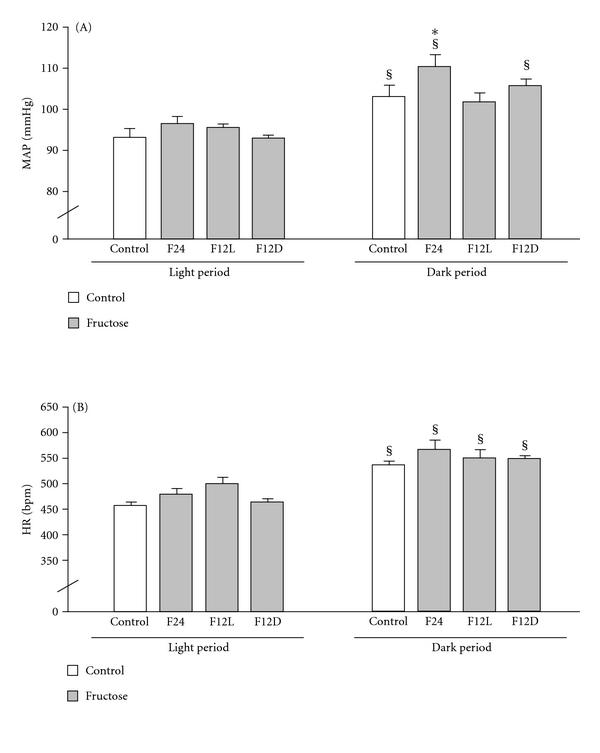
MAP (A) and HR (B) were recorded for 24 h and analyzed during 24 h light and dark phases. ANOVA showed main effect of light/dark for MAP (F (1.32) = 11.2, *P* < 0.005) and HR (F (1.32) = 16.62, *P* < 0.0003). ^§^ *P* < 0.05 light versus dark. *P < 0.05 versus Control.

Light/dark differences in BP were present in Control, F24, and F12D groups (Figure 5(A), *P* < 0.05). The circadian BP rhythm was not seen in the F12L (Figure 5(A)). Fructose consumption produced an increase in BP in the F24 group during the dark phase when compared to the Control group during the same phase (Figure 5(A), *P* < 0.05) and also when compared to F24 during the light phase (Figure 5(A), *P* < 0.05). This fructose effect was not observed in the F12D group (Figure 5(A)). All groups showed elevated HR during the dark as compared to the light phase (Figure 5(B), *P* < 0.05) with no effect of the fructose regimen (Figure 5(B)).

## 4. Discussion

The increase in fructose consumption, due to its widespread presence in the modern diet, has become a major public health concern. This is related to evidence that excessive fructose intake may have detrimental effects, including the promotion of obesity and its cardiovascular and metabolic complications [1, 2]. Studies focused on the direct effects of fructose intake in animals and humans showed increased BP, dyslipidemia, insulin resistance, and glucose intolerance [3–8]. In addition to the detrimental effects of a high-fructose diet, the timing of consumption might have additional effects. Changes in circadian feeding patterns, such as the night eating syndrome (NES), can promote weight gain, hormonal changes, and psychological disorders in either in animals or humans [10–14, 24]. In this study, we evaluated the time effect (light or dark) of fructose consumption on BP, body weight, and metabolic and hormonal parameters. We observed that day or night restricted fructose consumption increased body weight gain and glycemia. Further changes were observed in mice submitted to light-restricted fructose access: glucose intolerance, exacerbated hyperglycemia, and a lack of circadian BP oscillation. These additional changes were observed after 8 weeks of treatment, suggesting that the chronic aspect of the restricted regimen itself might be responsible, at least partially, for these results.

The circadian BP rhythm refers to the daily variation in BP that in humans shows higher levels during day vs. night. Healthy subjects usually present a 10–20% decline in arterial BP during nighttime intervals which is called the “dipper” pattern [25]. Impaired nocturnal BP decline (nondipping) is a BP abnormality that is frequently seen among patients with diabetes [26–30]. In order to follow the L/D BP rhythm in our study, we used radiotelemetry for BP monitoring in conscious, undisturbed mice. 24 hr BP recordings showed, as expected, that control mice exhibited light/dark circadian oscillations with lower BP seen during the light period, the inactive period for mice [4, 31–33]. When fructose access was restricted to the light phase (F12L), the light/dark BP rhythm was nonexistent. This could be related, at least partly, to the drinking pattern since the F12L group also showed a lack of circadian oscillation in fluid intake, that is, similar levels during the light and dark periods. This indicates that animals spent more time, during the light phase, drinking and therefore awake and possibly active. Blood pressure circadian rhythm disruption may also be due to changes in sleep since it has been observed in diabetic patients, who wake up more frequently during the night (for instance, due to nocturia) [34]. Although sleep disruption may also be associated with stress [35], stress as documented by unchanged urinary corticosterone does not seem to be responsible for the observed cardiovascular and metabolic effects. Therefore, the disturbance in circadian BP pattern in the F12L group might be due to the timing of fructose ingestion (during the light phase) itself. Possibly, a higher fructose load or longer treatment could produce a shift in the BP circadian pattern in the F12L. However, these speculations do not affect the conclusion drawn from this group: fructose access restricted to the light phase produces disruption in BP rhythms.

The circadian pattern on HR light/dark oscillations, showing a rhythmic pattern of variation in HR during 24 hours characterized by higher HR in the dark (awake period) over the light period (sleep period), was not affected by any of the treatments. As previously shown by our group [4, 22], although nocturnal BP was increased in F24 when compared to controls, no further changes due to fructose were observed in HR.

Circadian changes, such as the nondipping BP pattern, are associated with diabetic micro- and macrovascular [36–41] complications and end-organ damage [42]. The circadian changes in BP and fluid intake observed in F12L could also be implicated in the impaired glucose metabolism (exacerbated hyperglycemia and glucose intolerance), exclusively observed in this group (F12L). Preliminary information suggests derangements in renal histology in mice with fructose access restricted to the light phase (Morris et al., unpublished data). Changes in circadian pattern of food consumption can lead to weight gain, metabolic dysfunction [10–14, 43], and increased risk of obesity and diabetes [44] in humans. The light phase-restricted fructose drinking regimen could be a parallel for human NES, which is associated with hyperglycemia, glucose intolerance [45], and weight gain without changes in total caloric consumption [12, 46], similar to our observations in mice. Although the mechanism behind light-fed weight gain in mice is unknown, the study of Arble et al., showed that there is causal evidence that feeding at the “wrong” time can lead to weight gain [10].

Although F12L mice showed greater impairment in glucose handling, both groups on restricted fructose access (F12L and F12D) showed increased BW gain and hyperglycemia, not observed in the F24 group. Chronic misalignments between endogenous circadian timing system and behavioral cycles have adverse metabolic effects in humans [43], including increased risk of obesity and diabetes [44]. As shown by our group [4, 6], 24 h access to fructose produces glucose intolerance without increases in BW. Therefore, the circadian restriction aspect of the drinking regimen itself could contribute to the increase in weight gain and blood glucose levels observed in F12L and F12D.

A high-fructose diet in rodents has been associated with cardiovascular dysregulation and insulin resistance [4, 6, 47–49], More specifically, increased fructose intake, in both, animals and humans, increases blood pressure and causes dyslipidemia and changes in glucose handling [3–8]. As expected, mice receiving fructose 24 h a day exhibited nocturnal hypertension [4, 6]. However, BP was not increased in groups on restricted fructose access (F12L and F12D) despite the circadian disruption observed in the F12L group. Diet concentration and length of treatment are important factors for hypertension development in the rodent fructose model [50]. Therefore, the restricted aspect of the fructose access regimen itself could be a possible explanation for the absence of BP increase in the F12L and F12D groups.

As mentioned previously, fructose consumption is often associated with dyslipidemia. Chronic consumption of fructose led to hypercholesterolemia without changes in plasma triglycerides [4, 6, 51, 52]. However, in this study, the F24 group showed no changes in cholesterol. This could be due to the nature of the fructose source, since in previous studies [4, 6] fructose was given in the chow (pellet diet containing 67% carbohydrate—98% fructose, 13% fat, and 20% protein) instead of as a fluid (10% fructose solution). Both groups on restricted access (F12L and F12D) had lower levels of triglycerides with no changes in cholesterol. Since circulating factors such as glucose and triglycerides can be modulated by time-restricted food intake access [13, 53], the restricted nature of the fructose drinking regimen itself could explain the results observed in these groups.

## 5. Conclusion

In conclusion, we demonstrated that circadian phase restriction of fructose access leads to changes in glucose homeostasis, body weight gain, and light/dark BP rhythms. The data suggests that even moderate circadian changes might contribute to the onset or development of the metabolic syndrome symptoms, which might be exacerbated by the timing of fructose consumption. The results have clinical implications since time of day and intake may be considered in the overall treatment regimen.

## Figures and Tables

**Table 1:** Plasma insulin, triglycerides, and cholesterol and urinary corticosterone.

	Control	F24	F12 L	F12 D
Insulin (ng/mL)	0.6 ± 0.1	0.8 ± 0.1	0.5 ± 0.1	1.2 ± 0.3
TGL (mg/dL)	104 ± 18	129 ± 13	85 ± 1^#^	89 ± 11^#^
Cholesterol (mg/dL)	95 ± 13	94 ± 11	94 ± 11	81 ± 13
Corti (mg/mmol/L)	0.24 ± 0.1	0.36 ± 0.1	0.36 ± 0.1	0.23 ± 0.1

Plasma insulin, triglycerides, and cholesterol levels were measured in nonfasted mice at 8-9 wk. ANOVA showed a significant effect of diet for insulin (F (3.17) = 3.45, *P* < 0.05) and triglycerides (F (3.17) = 2.95, *P* < 0.05). ^#^ *P* < 0.01 vs. F24.

**Table 2:** Effect of restricted fructose access on fluid intake.

	Water (Control)	F24	F12L	F12D
24 h volume (mL)	11.5 ± 0.7	20.2 ± 2.4*	11.5 ± 1.6	19.8 ± 1.5*
Light phase (%)	30.4 ± 6.0	32.8 ± 3.9	44.0 ± 1.8*	25.4 ± 5.6
Dark phase (%)	69.6 ± 6.0^#^	67.2 ± 3.9^#^	56.0 ± 1.8	74.6 ± 5.6^#^

ANOVA showed a significant effect of diet for 24 h volume intake [F (3.21) = 8.9, *P* < 0.001]. ANOVA showed main effect of light/dark [F (1.42) = 100.6, *P* < 0.00001] and interaction between light/dark and diet [F (3.42) = 5.6, *P* < 0.003]. *P < 0.01 vs. Control. ^#^ *P* < 0.01 vs. light.

## References

[1] Elliott SS, Keim NL, Stern JS, Teff K, Havel PJ (2002). Fructose, weight gain, and the insulin resistance syndrome. *The American Journal of Clinical Nutrition*.

[2] Bray GA, Nielsen SJ, Popkin BM (2004). Consumption of high-fructose corn syrup in beverages may play a role in the epidemic of obesity. *The American Journal of Clinical Nutrition*.

[3] Brown CM, Dulloo AG, Yepuri G, Montani JP (2008). Fructose ingestion acutely elevates blood pressure in healthy young humans. *American Journal of Physiology*.

[4] Farah V, Elased KM, Chen Y (2006). Nocturnal hypertension in mice consuming a high fructose diet. *Autonomic Neuroscience*.

[5] Furuhashi M, Ura N, Takizawa H (2004). Blockade of the renin-angiotensin system decreases adipocyte size with improvement in insulin sensitivity. *Journal of Hypertension*.

[6] Senador D, Key M, Brosnihan KB, Irigoyen MC, Elased KM, Morris M (2010). Cardiovascular interactions between losartan and fructose in mice. *Journal of Cardiovascular Pharmacology and Therapeutics*.

[7] Shinozaki K, Ayajiki K, Nishio Y, Sugaya T, Kashiwagi A, Okamura T (2004). Evidence for a causal role of the renin-angiotensin system in vascular dysfunction associated with insulin resistance. *Hypertension*.

[8] Teff KL, Grudziak J, Townsend RR (2009). Endocrine and metabolic effects of consuming fructose- and glucose-sweetened beverages with meals in obese men and women: influence of insulin resistance on plasma triglyceride responses. *Journal of Clinical Endocrinology and Metabolism*.

[9] de Angelis K, Senador DD, Mostarda CT, Irigoyen MC, Morris M Sympathetic overactivity precedes metabolic dysfunction in a fructose model of glucose intolerance in mice.

[10] Arble DM, Bass J, Laposky AD, Vitaterna MH, Turek FW (2009). Circadian timing of food intake contributes to weight gain. *Obesity*.

[11] Bodosi B, Gardi J, Hajdu I, Szentirmai E, Obal F, Krueger JM (2004). Rhythms of ghrelin, leptin, and sleep in rats: effects of the normal diurnal cycle, restricted feeding, and sleep deprivation. *American Journal of Physiology*.

[12] Colles SL, Dixon JB, O’Brien PE (2007). Night eating syndrome and nocturnal snacking: association with obesity, binge eating and psychological distress. *International Journal of Obesity*.

[13] Kudo T, Akiyama M, Kuriyama K, Sudo M, Moriya T, Shibata S (2004). Night-time restricted feeding normalises clock genes and Pai-1 gene expression in the db/db mouse liver. *Diabetologia*.

[14] Zvonic S, Ptitsyn AA, Conrad SA (2006). Characterization of peripheral circadian clocks in adipose tissues. *Diabetes*.

[15] Åkerstedt T, Kecklund G, Johansson SE (2004). Shift work and mortality. *Chronobiology International*.

[16] Scheer FAJL, Hilton MF, Mantzoros CS, Shea SA (2009). Adverse metabolic and cardiovascular consequences of circadian misalignment. *Proceedings of the National Academy of Sciences of the United States of America*.

[17] Ando H, Kumazaki M, Motosugi Y (2011). Impairment of peripheral circadian clocks precedes metabolic abnormalities in ob/ob mice. *Endocrinology*.

[18] Okamura H, Doi M, Yamaguchi Y, Fustin J-M (2011). Hypertension due to loss of clock: novel insight from the molecular analysis of *Cry1/Cry2*-deleted mice. *Current Hypertension Reports*.

[19] Hu FB (2011). Globalization of diabetes: the role of diet, lifestyle, and genes. *Diabetes Care*.

[20] Hofmann SM, Tschöp MH (2009). Dietary sugars: a fat difference. *Journal of Clinical Investigation*.

[21] Lakka TA, Bouchard C (2005). Physical activity, obesity and cardiovascular diseases. *Handbook of Experimental Pharmacology*.

[22] Farah V, Elased KM, Morris M (2007). Genetic and dietary interactions: role of angiotensin AT_1a_ receptors in response to a high-fructose diet. *American Journal of Physiology*.

[23] Wichi RB, Farah V, Chen Y, Irigoyen MC, Morris M (2007). Deficiency in angiotensin AT_1a_ receptors prevents diabetes-induced hypertension. *American Journal of Physiology*.

[24] Morris M, Araujo IA, Pohlman RL, Marques MC, Rodwan NS, Farah V (2012). Timing of fructose intake: an important regulator of adiposity. *Clinical and Experimental Pharmacology and Physiology*.

[25] Pierdomenico SD, Lapenna D, Guglielmi MD (1997). Arterial disease in dipper and nondipper hypertensive patients. *American Journal of Hypertension*.

[26] Chau NP, Bauduceau B, Chanudet X, Larroque P, Gautier D (1994). Ambulatory blood pressure in diabetic subjects. *American Journal of Hypertension*.

[27] Fogari R, Zoppi A, Malamani GD, Lazzari P, Destro M, Corradi L (1993). Ambulatory blood pressure monitoring in normotensive and hypertensive type 2 diabetics: prevalence of impaired diurnal blood pressure patterns. *American Journal of Hypertension*.

[28] Ikeda T, Matsubara T, Sato Y, Sakamoto N (1993). Circadian blood pressure variation in diabetic patients with autonomic neuropathy. *Journal of Hypertension*.

[29] Rutter MK, McComb JM, Forster J, Brady S, Marshall SM (2000). Increased left ventricular mass index and nocturnal systolic blood pressure in patients with type 2 diabetes mellitus and microalbuminuria. *Diabetic Medicine*.

[30] Wiegmann TB, Herron KG, Chonko AM, MacDougall ML, Moore WV (1990). Recognition of hypertension and abnormal blood pressure burden with ambulatory blood pressure recordings in type I diabetes mellitus. *Diabetes*.

[31] Chen D, La LG, Head GA, Walther T, Mayorov DN (2010). The day-night difference of blood pressure is increased in AT_1A_-receptor knockout mice on a high-sodium diet. *American Journal of Hypertension*.

[32] Sheward WJ, Naylor E, Knowles-Barley S (2010). Circadian control of mouse heart rate and blood pressure by the suprachiasmatic nuclei: behavioral effects are more significant than direct outputs. *PloS ONE*.

[33] Senador D, Kanakamedala K, Irigoyen MC, Morris M, Elased KM (2009). Cardiovascular and autonomic phenotype of db/db diabetic mice. *Experimental Physiology*.

[34] Perk G, Mekler J, Ben ID, Bursztyn M (2002). Non-dipping in diabetic patients: insights from the siesta. *Journal of Human Hypertension*.

[35] Heinrichs SC, Koob GF Application of experimental stressors in laboratory rodents. *Current Protocols in Neuroscience*.

[36] Hansen HP, Rossing P, Tarnow L, Nielsen FS, Jensen BR, Parving HH (1996). Circadian rhythm of arterial blood pressure and albuminuria in diabetic nephropathy. *Kidney International*.

[37] Knudsen SO, Poulsen PL, Hansen KWÜ, Ebbehøj E, Bek T, Mogensen CE (2002). Pulse pressure and diurnal blood pressure variation: association with micro- and macrovascular complications in type 2 diabetes. *American Journal of Hypertension*.

[38] Knudsen ST, Jeppesen P, Frederiksen CA (2007). Endothelial dysfunction, ambulatory pulse pressure and albuminuria are associated in type 2 diabetic subjects. *Diabetic Medicine*.

[39] Nakano S, Fukuda M, Hotta F (1998). Reversed circadian blood pressure rhythm is associated with occurrences of both fatal and nonfatal vascular events in NIDDM subjects. *Diabetes*.

[40] Nakano Y, Oshima T, Ozono R (2001). Non-dipper phenomenon in essential hypertension is related to blunted nocturnal rise and fall of sympatho-vagal nervous activity and progress in retinopathy. *Autonomic Neuroscience*.

[41] Nielsen FS, Rossing P, Bang LE (1995). On the mechanisms of blunted nocturnal decline in arterial blood pressure in NIDDM patients with diabetic nephropathy. *Diabetes*.

[42] Spallone V, Maiello MR, Cicconetti E (2001). Factors determining the 24-h blood pressure profile in normotensive patients with type 1 and type 2 diabetes. *Journal of Human Hypertension*.

[43] Kohsaka A, Bass J (2007). A sense of time: how molecular clocks organize metabolism. *Trends in Endocrinology and Metabolism*.

[44] Kroenke CH, Spiegelman D, Manson J, Schernhammer ES, Colditz GA, Kawachi I (2007). Work characteristics and incidence of type 2 diabetes in women. *American Journal of Epidemiology*.

[45] Qin LQ, Li J, Wang Y, Wang J, Xu JY, Kaneko T (2003). The effects of nocturnal life on endocrine circadian patterns in healthy adults. *Life Sciences*.

[46] Allison KC, Ahima RS, O’Reardon JP (2005). Neuroendocrine profiles associated with energy intake, sleep, and stress in the night eating syndrome. *Journal of Clinical Endocrinology and Metabolism*.

[47] Dai S, Todd ME, Lee S, McNeill JH (1994). Fructose loading induces cardiovascular and metabolic changes in nondiabetic and diabetic rats. *Canadian Journal of Physiology and Pharmacology*.

[48] Delbosc S, Paizanis E, Magous R (2005). Involvement of oxidative stress and NADPH oxidase activation in the development of cardiovascular complications in a model of insulin resistance, the fructose-fed rat. *Atherosclerosis*.

[49] Hwang I, Ho H, Hoffman BB, Reaven GM (1987). Fructose-induced insulin resistance and hypertension in rats. *Hypertension*.

[50] Dai S, McNeill JH (1995). Fructose-induced hypertension in rats is concentration- and duration-dependent. *Journal of Pharmacological and Toxicological Methods*.

[51] Ostos MA, Recalde D, Baroukh N (2002). Fructose intake increases hyperlipidemia and modifies apolipoprotein expression in apolipoprotein AI-CIII-AIV transgenic mice. *Journal of Nutrition*.

[52] Nagata R, Nishio Y, Sekine O (2004). Single nucleotide polymorphism (-468 Gly to Ala) at the promoter region of sterol regulatory element-binding protein-1c associates with genetic defect of fructose-induced hepatic lipogenesis. *Journal of Biological Chemistry*.

[53] Oishi K, Ohkura N, Kasamatsu M (2004). Tissue-specific augmentation of circadian PAI-1 expression in mice with streptozotocin-induced diabetes. *Thrombosis Research*.

